# Color Flow Doppler Echocardiography in Healthy Racing Pigeons (*Columba livia* f. domestica) and the Evidence of Physiological Blood Flow Vortex Formations

**DOI:** 10.3390/vetsci7020060

**Published:** 2020-05-04

**Authors:** Marko Legler, Lajos Koy, Norbert Kummerfeld, Michael Fehr

**Affiliations:** Clinic for Small Mammals, Reptiles and Birds, University of Veterinary Medicine Hannover, Foundation, Bünteweg 9, D-30559 Hannover, Germany; lajos.schmitt@gmx.de (L.K.); nokumf@gmx.de (N.K.); Michael.Fehr@tiho-hannover.de (M.F.)

**Keywords:** color Doppler sonography, heart, blood flow vortices, diastole, systole, birds, valva pulmonis

## Abstract

In avian medicine, Doppler sonographic techniques are used to visualize and estimate blood flow in the heart. In the literature there is a lack of standardized studies of the use of color Doppler flow on healthy avian species. For this purpose, we examined blood flow in the heart in the four-chamber view of clinically healthy awake racing pigeons (*n* = 43) by color flow Doppler sonography. With this technique the diastolic and systolic blood flow in the heart chambers and the heart valve regions were well visualized. However, the pulse repetition frequency must be adapted to the specific blood flow velocities of the heart region to be measured to reduce aliasing in higher velocities and to visualize blood flow of lower velocities. With the help of color Doppler imaging in the four-chamber view, typical physiological atrial and ventricular blood flow vortex formations were visualized in the avian heart for the first time. In the left ventricle an asymmetric vortex ring in the passive and active ventricular filling, in the right ventricle a great counter-clockwise blood vortex in the active ventricular filling, in the left atrium a vortex clockwise, and in the right atrium counter-clockwise were observed. The knowledge of these physiological blood flow vortices is important to identify pathological blood flow.

## 1. Introduction

The four-chambered avian heart is functionally comparable to the mammalian heart, with a left and right ventricle and two atria [1,2,3,4]. Unique to avian species is the anatomy of the atrioventricular (AV) valves. The left AV valve is formed as a membranous tricuspid AV valve with a longer septal cusp, similar to the anatomical structure of mammalian species. In contrast, the right AV valve is an oblique muscular flap of the right ventricular free wall and consists of atrial and ventricular myocardial parts [1,2,5]. The function of the left AV valve is a passive closure of the AV orifice depending on the pressure gradient. In contrast, the muscular structure of the right AV valve allows an active closure of the right AV orifice and participation of this valve in the pumping function of the right ventricle [5,6]. To fulfil this special function the right valve is separately innervated by an atrioventricular ring bundle of pacemaker and conducting myocytes (formerly known as AV-Purkinje ring) an important part of the conducting system of the avian heart [7,8,9]. The aortic and pulmonary valves consist of three semilunar cusps that work passively depending on the pressure gradient [1,2].

Doppler flow imaging is an important tool in human and small animal cardiology to visualize and measure blood flow in the heart, to detect turbulent flow, and to diagnose pathological conditions [10,11,12,13]. In avian medicine Doppler sonographic techniques are also used to visualize and estimate blood flow in the heart [3,4,14,15,16,17,18,19,20,21,22,23]. In particular, color flow Doppler imaging is used in avian medicine to visualize heart valve insufficiencies, aneurysm, and atrial or ventricular septal defects [3,4]. Small valvular regurgitations are easier to diagnose in color flow sonography compared to pulse wave (PW) or continued wave (CW) Doppler imaging [23]. In color flow images velocity information is combined with anatomical information. Movements away from the transducer are normally coded in a blue color and movements toward the transducer are colored in red. The use of the color flow Doppler sonography is described in the literature in birds mainly in clinical cases and there is a lack of standardized studies on healthy animals [3,4,14]. However, in the use of Doppler echocardiography it is important to distinguish physiological flow pattern from a pathological turbulent flow. The aim of the present study is to examine the blood flow in the heart of clinically healthy awake racing pigeons by color flow Doppler sonography to verify the visualization of the blood flow and to determine the function of the heart valves.

## 2. Materials and Methods

The study was conducted in accordance with the German animal welfare regulations and with the permission of the relevant German authorities (reference number: 33.12-42502-04-15/1864).

### 2.1. Experimental Animals

Racing pigeons (*Columba livia* f. dom.; *n* = 43) of both sexes (male: *n* = 16; female: *n* = 27) were used for the investigation. The pigeons were 2.30 ± SD 1.69 (range: 0.5 to 8) years old and had a weight of 468.16 g ± SD 51.64 (range: 352–577 g) body mass and were trained for racing. The sternal length of the pigeons measured from the visible sternocoracoid joint to the end of the sternum in laterolateral radiographic images was 73.4 mm ± SD 3.1 mm (range: 63.2–81.0 mm). The pigeons were housed in indoor aviaries, offered a commercial pigeon seed mix and fresh drinking water ad libitum, and routinely vaccinated by the owner for pigeon avulavirus 1 (paramyxovirus 1) and salmonellosis. All pigeons showed normal feeding and drinking behavior. Prior to the ultrasound examinations the pigeons were acclimatized for two weeks in the new aviaries. In this time a bacteriological and parasitological testing of a composite fecal sample of the pigeons for salmonella and endoparasites of the intestine were negative. A microscopic examination of fresh crop samples revealed in some pigeons a low-grade infestation with *Trichomonas gallinae* and all pigeons were treated with 10 mg carnidazole (Spatrix^®^, Elanco Deutschland GmbH, Bad Homburg, Germany). The pigeons were declared healthy by clinical, hematological, and radiological examinations. The maximum width of the cardiac silhouette of the pigeons measured in the ventrodorsal radiograph was 58.8% ± 3.3 (50.3%–65.1%) of the maximum width of the thorax. These results are comparable to results of healthy birds in the literature [3]. The hematocrit (44.9% ± 1.8; 42.0%–49.0%) and buffy coat (<1%) of the pigeons were in the reference values of healthy and normal hydrated pigeons [24].

Egg-laying pigeons were excluded from sonographic examinations to prevent the influence of a higher abdominal pressure.

### 2.2. Doppler-Sonographic Examination

Echocardiographic images were acquired by using a 10 MHz phased-array transducer (GE 10S-RS Probe; B Mode 4.5–11.5 MHz) with a digital ultrasound system (Vivid 7 Dimension BT08, GE Medical Systems) in combination with an electrocardiogram (ECG) according to Einthoven [25,26,27]. The pigeons were fixed in a semi-upright position for the sonographic examination of the heart from the left and right parasternal approach. Depending on the individual bird, the left and right fenestra or the space behind the last rib through the liver to the heart were chosen as acoustic windows. The 2D echocardiographic images were named and oriented in accordance with Pees et al. [3], Riedel [28], Schulz [29]. The right and left parasternal longitudinal horizontal views (“four-chamber view”) were chosen individually to visualize the left and right heart ventricle, the AV valves, and the atria as well as the aorta with aortic valve and, after horizontal correction of the transducer, the right heart outflow tract and pulmonary artery. The 2D images were overlaid with color flow Doppler information of the ultrasound system. The field with color flow Doppler information was chosen as small as possible to guarantee frame rates above 80 frames per second (125.5 FPS ± SD 30.2). The pulse repetition frequency (PRF) value was adapted individually to the anticipated blood flow velocities between 1 and 11.76 kHz to reduce aliasing. Depending on the PRF, a wall filter of 6.54 to 19.63 cm per second (15.91 cm s^−1^ ± SD 2.99) was used. The sample volume setting was 0.4 mm. Flow towards the transducer was color-coded in red and flow away from the transducer was color-coded in blue. The sonographic examinations were stored on the ultrasound system and evaluated in slow motion.

The color flow patterns within the chambers and heart valves were analyzed in a qualitative manner relative to anatomic location and timing within the cardiac cycle.

### 2.3. Statistical Analysis

The blood flow in the color Doppler images was described and the results expressed as the percentage of total number of examined pigeons. For the comparison of the blood flow of different anatomical structures the Chi-squared test was implemented. Mean, standard deviation (SD), range (Xmin to Xmax) and median were calculated for the used PRF. The Kolmogorov–Smirnov test was used to test for normal distribution. According to these results the Mann–Whitney U test was chosen for the evaluations. Statistical tests were performed using SPSS^®^ Statistics 26. A significance level of *p* ≤ 0.05 was chosen.

## 3. Results

Adequate Doppler sonographic examinations of the diastolic and systolic blood flow were possible in all 43 racing pigeons in the heart rate range of 220.5 ± 41.3 heart beats per minute (left heart) and 219.1 ± 34.7 heart beats per minute (right heart). A fused diastolic ventricular inflow (fused E and A wave, EA wave) was observed in five birds in a heart rate range of 270 to 360 heart beats per minute. The significant differences between the diastolic and systolic blood flow velocities led to different sonographic settings (PRF) for the visualization of these different blood flows. For the examination of the diastolic blood flow of the left heart a PRF of 6.35 kHz ± SD 1.25 (4.0 to 9.0 kHz; 6.0 kHz; color scale: 0.52 m s^−1^ ± SD 0.10; 0.33 to 0.74 m s^−1^; 0.49 m s^−1^) and of the right heart a PRF of 5.94 kHz ± SD 1.62 (2.0 to 9.0 kHz; 6.0 kHz; color scale: 0.48 m s^−1^ ± SD 0.13; 0.16 to 0.74 m s^−1^; 0.49 m s^−1^) were used (no significant difference Mann–Whitney U test *p* = 0.17). The blood flow in the atria was also examined with these settings. For the examination of the systolic blood flow of the pulmonary artery a PRF of 8.87 kHz ± SD 1.71 (5.0 to 11.76 kHz; 9.0 kHz; color scale: 0.73 m s^−1^ ± SD 0.14; 0.41 to 0.98 m s^−1^; 0.74) and of the aorta a PRF of 9.46 kHz ± SD 1.77 (6.0 to 11.76 kHz; 10.0 kHz; color scale: 0.79 m s^−1^ ± SD 0.15; 0.49 to 0.98 m s^−1^; 0.82 m s^−1^) was used (no significant difference Mann–Whitney U test *p* = 0.07). In contrast to this, the PRF values used to visualize the diastolic and systolic blood flow are highly significantly different (*p* < 0.001; Mann–Whitney U test).

### 3.1. Left Ventricular Filling

**Figure 1:**
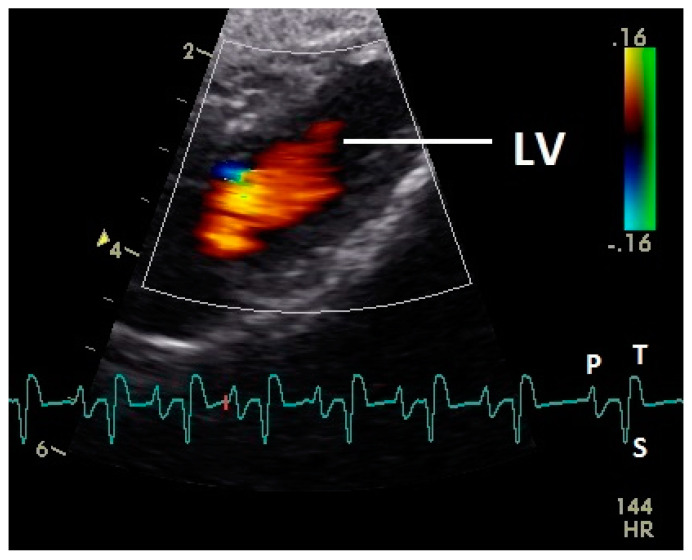
Color Doppler image of the early diastolic filling of the left ventricle. The blood inflow in the left ventricle from the atrium (red signal) and vortex formation of the blood flow behind the septal part of the left atrioventricular (AV) valve leaflet and left outflow tract (blue signal) is visible. LV: left ventricle; HR heart rate. Electrocardiogram: P: P wave, S: S wave, T: T wave. The color scale on the right of the image is calibrated in m s^−1^.

**Figure 2:**
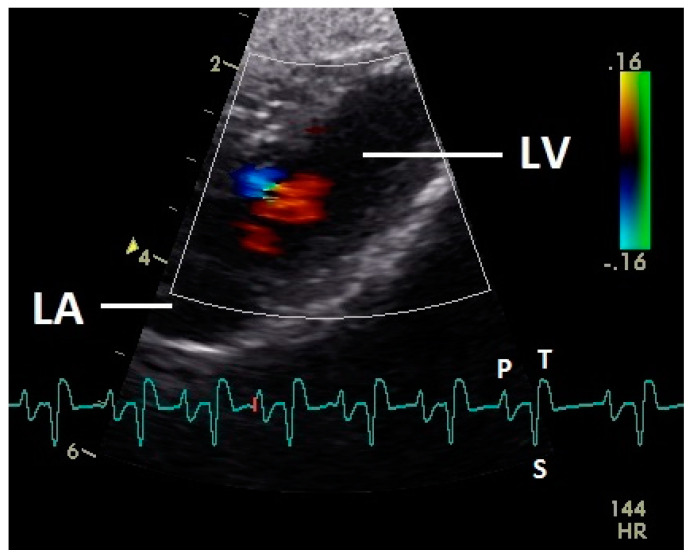
Color Doppler image of the ventricle in mid diastolic phase. The vortex formation of the blood in the ventricle is visible (red and blue signal). LV: left ventricle; LA: left atrium; HR: heart rate. Electrocardiogram: P: P wave, S: S wave, T: T wave. The color scale on the right of the image is calibrated in m s^−1^.

**Figure 3:**
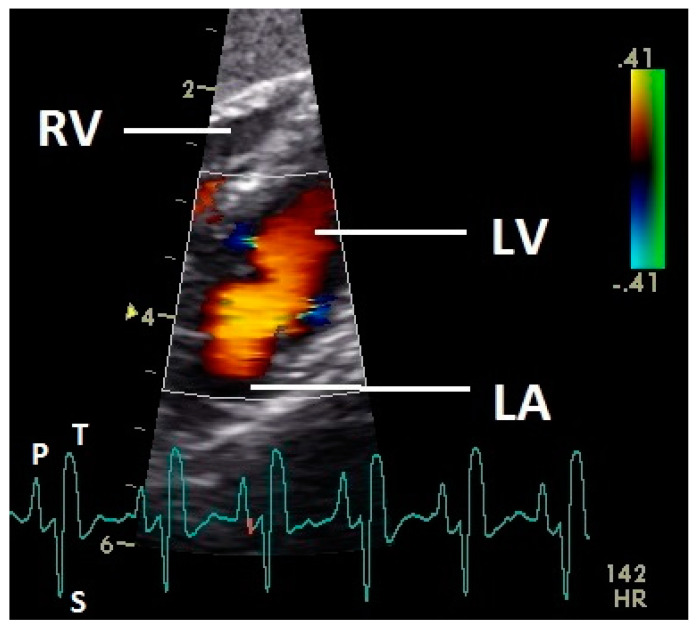
Color Doppler image of the late active filling of the left ventricle. The blood inflow in the left ventricle from the atrium (red signal) and vortex formation of the blood flow behind the septal part of the left AV valve leaflet, left outflow tract, and the smaller lateral leaflets of the left AV valve (blue signals) are visible. LV: left ventricle; LA: left atrium; RV: right ventricle; HR: heart rate. Electrocardiogram: P: P wave, S: S wave, T: T wave. The color scale on the right of the image is calibrated in m s^−1^.

Diastolic ventricular inflow was recorded in the atrium, across the AV valve and within the ventricular inlets and outlets. In the early diastole a red signal was recorded within the left atrium and across the mitral valve annulus into the left ventricle. This phase was interpreted as the passive ventricular filling due to left ventricular relaxation (descending part of T wave in ECG). Behind the longer septal part of the left AV valve leaflet and in the left outflow tract a blue signal in this phase of the cardiac cycle simultaneous to the red signal was observed in 42 birds (97.7%; Figure 1) and in the area of the lateral smaller parts of the left AV valve only in nine birds (20.9%; significant difference Chi-squared test *p* ≤ 0.001). In the mid diastolic phase in some pigeons the movement of the blood in the ventricle was observed (Figure 2). Within the P wave of the ECG an intense red blood flow signal was evident in the left atrium, crossing the left AV valve and entering the left ventricle as a sign of blood flow caused by atrial contraction. This rapid ventricular filling led to a visible blue signal in the left outflow tract, behind the septal part of the AV valve (97.7%), and in the some pigeons (48.8%; significant difference Chi-squared test *p* ≤ 0.001) also behind the smaller lateral leaflets of the left AV valve simultaneous to the red signal (Figure 3). In one pigeon the passive diastolic inflow was connected with the active ventricular filling (EA wave). 

### 3.2. Left Ventricular Ejection

**Figure 4:**
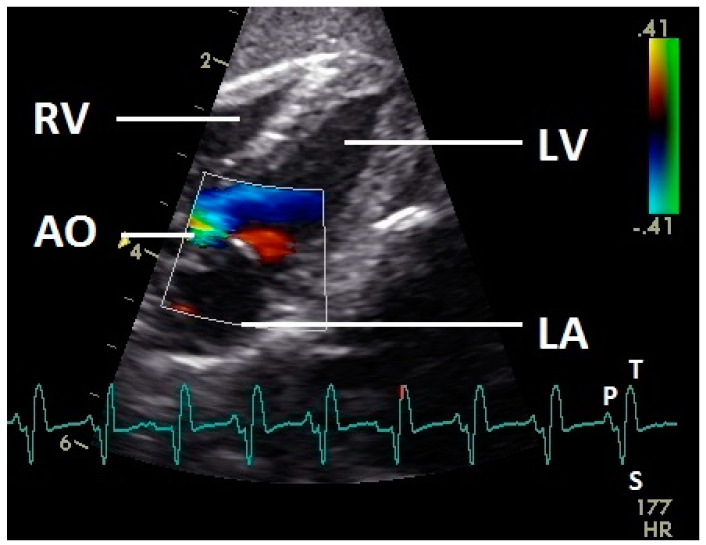
Color Doppler image of the left ventricle in the systole. The blood ejection from the left ventricle in the aorta is visible (blue signal). The blue flow signal is aliased in the aorta (wrong direction: red instead of blue of the color Doppler signal in the aorta). The movement of the valve level of the heart and the resulting blood flow are visible as a red signal. LV: left ventricle; LA: left atrium; AO: aorta; RV: right ventricle; HR: heart rate. Electrocardiogram: P: P wave, S: S wave, T: T wave. The color scale on the right of the image is calibrated in m s^−1^.

Left ventricular ejection, displayed as a blue flow signal, was observed during systole (ascending part of S wave to descending part of T wave of ECG) in the ventricle, left ventricular outflow tract, and aorta. This blue flow signal was aliased in all pigeons especially in the aorta with our settings. In the first part of the systole in all pigeons a simultaneous red ventricular blood flow signal to the much larger blue signal under the AV (Figure 4) valve was observed. During the closure of the aortic valve a little red signal in the area of this heart valve was realized in some pigeons (*n* = 11).

In the preejection period, between the end of the active diastolic filling and the contraction of the ventricle myocardium, we found in two pigeons (4.7%) a blue blood flow signal between the leaflets of the left AV valve to the atrium, a sign of an insufficiency of the left AV valve in this heart phase.

### 3.3. Left Atrial Filling

**Figure 5:**
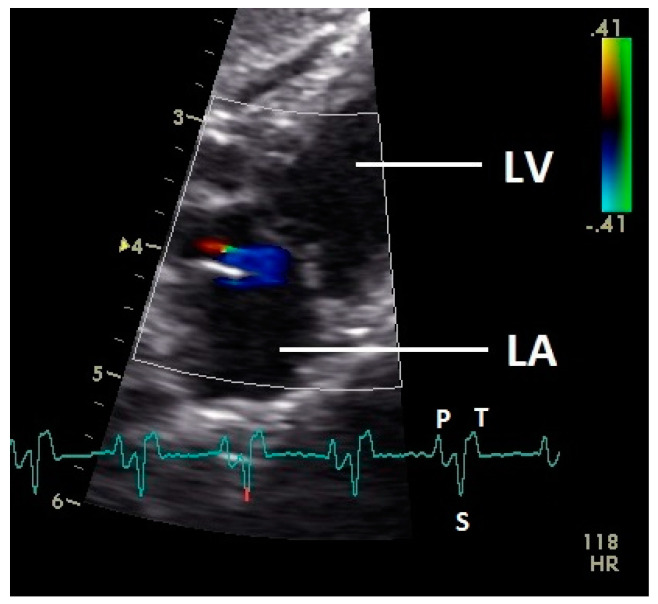
Color Doppler image of the early blood inflow in the left atrium (red and blue signal). LV: left ventricle; LA: left atrium; HR: heart rate. Electrocardiogram: P: P wave, S: S wave, T: T wave. The color scale on the right of the image is calibrated in m s^−1^.

**Figure 6:**
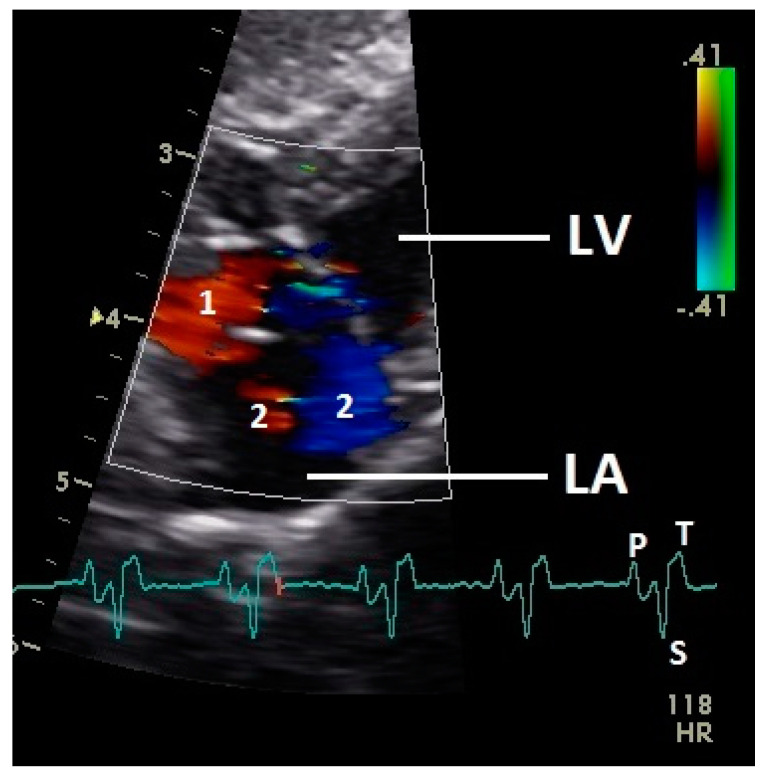
Color Doppler image of the late blood inflow in the left atrium (red signal; 1) and blood flow vortex formation (2). LV: left ventricle; LA: left atrium; HR: heart rate. Electrocardiogram: P: P wave, S: S wave, T: T wave. The color scale on the right of the image is calibrated in m s^−1^.

**Figure 7:**
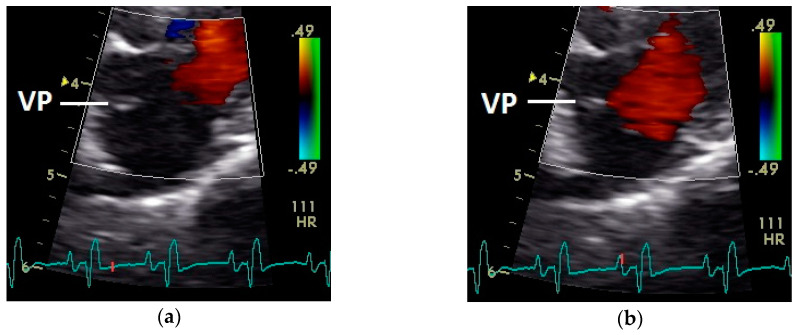
Position of the valva pulmonis (VP) in the diastole: (**a**) passive and (**b**) active ventricular filling (red signal); HR: heart rate; color scale of the color Doppler image is calibrated in m s^−1^.

After the P wave of the ECG in the phase of the relaxation of myocardium of the left atrium, a little blue filling signal over the AV valve was seen in the some pigeons (*n* = 27; 62.8%) with the used settings (Figure 5). In all pigeons a later and faster blue inflow signal in the atrium in the time of the descending part of the T wave was recorded over the AV valve to the left part of the atrium (significant difference Chi-squared test *p* ≤ 0.001). At the same time a red signal was seen right and left from the valva pulmonis in some pigeons (25 out of 43 birds; 58.1%; Figure 6). The position of the valva pulmonis was almost constant in diastole (Figure 7) and systole (Figure 5 and Figure 6).

### 3.4. Right Ventricular Filling

**Figure 8:**
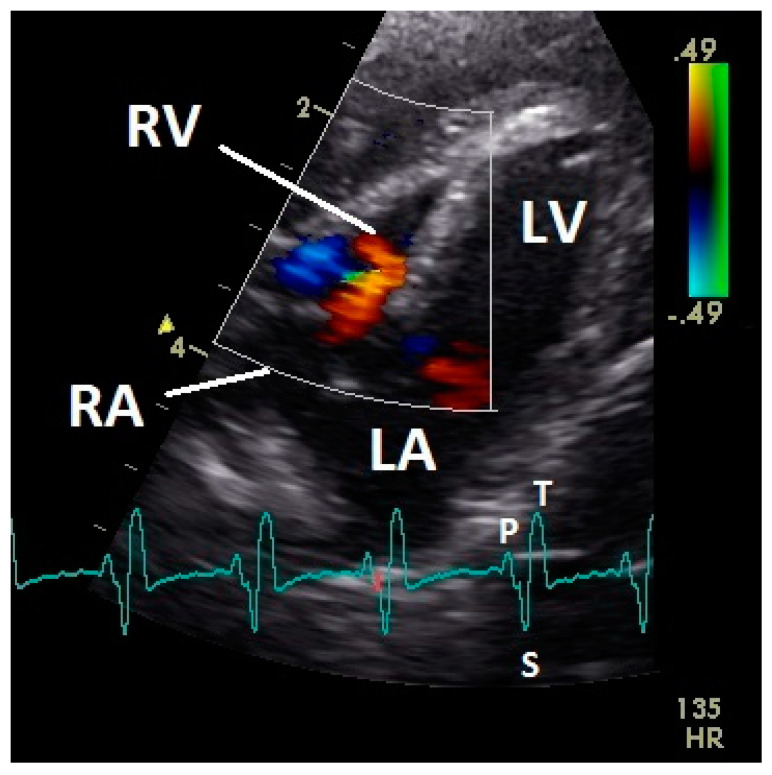
Color Doppler image of the late active filling of the right ventricle. The blood inflow in the right ventricle from the atrium (red signal) and vortex formation of the blood flow behind the right muscular AV valve (blue signal) are visible. LV: left ventricle; LA: left atrium; RV: right ventricle; RA: right atrium; HR: heart rate. Electrocardiogram: P: P wave, S: S wave, T: T wave. The color scale on the right of the image is calibrated in m s^−1^.

The early passive diastolic inflow in the right ventricle was visible as a red blood flow signal in 42 pigeons (97.7%; descending part of T wave in ECG). In four pigeons the passive diastolic inflow was connected with the active ventricular filling (EA wave). In the mid diastolic phase of the heart cycle no blood flow was recorded. During the active ventricular filling of the right ventricle (P wave of ECG) an intense red signal of high velocity was visible in all pigeons. In the active filling of the right ventricle, an intense blue signal behind the muscular right AV valve simultaneous to the larger red signal was detected in all pigeons (Figure 8). For some pigeons (25.6%) in this phase of the ventricular filling in the septal area of the blood inflow, a blue signal was also visible (*p* ≤ 0.001; Chi-squared test).

### 3.5. Right Atrial Filling

**Figure 9:**
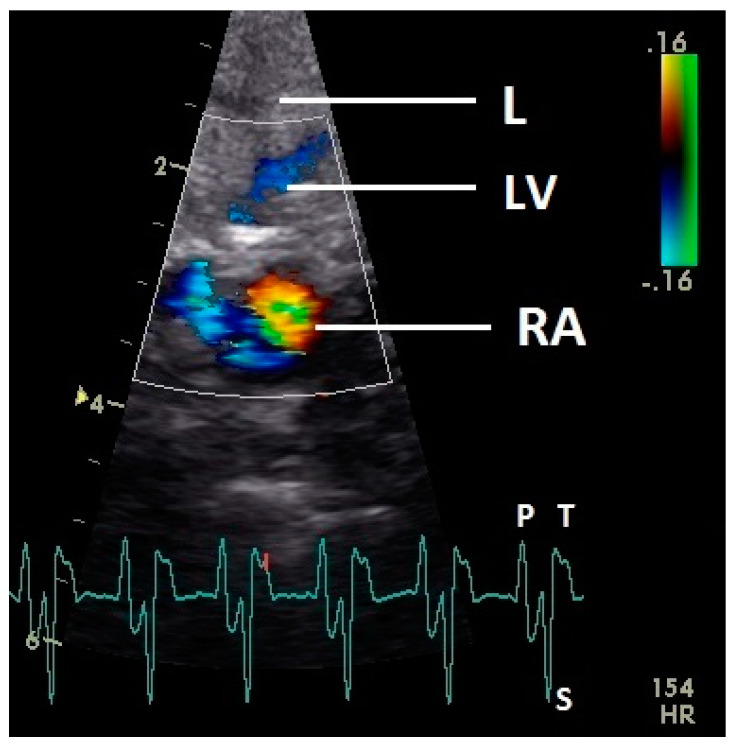
Color Doppler image of the late blood inflow in the right atrium (blue signal). The vortex formation of the blood flow in the atrium (red and blue signal simultaneously) and the blood flow in the liver vein (LV; blue signal in liver tissue) are visible. RV: right ventricle; RA: right atrium; HR: heart rate. Electrocardiogram: P: P wave, S: S wave, T: T wave. The color scale on the right of the image is calibrated in m s^−1^.

The blood flow in the right atrium was observed in 35 pigeons and was difficult to examine with our settings. After the P wave of the ECG in the phase of the relaxation of the myocardium of the right atrium, a blue filling signal in 15 pigeons (34.9%) was visualized. In the second half of the systole (T wave) a late blue filling inflow signal in the atrium was seen in 35 pigeons (81.4%); at this time a blood flow in the liver veins was also observed (Figure 9). Simultaneously to the blue inflow signal a red blood flow signal in the area of the septum in the right atrium was observed (Figure 9).

### 3.6. Right Ventricular Ejection

**Figure 10:**
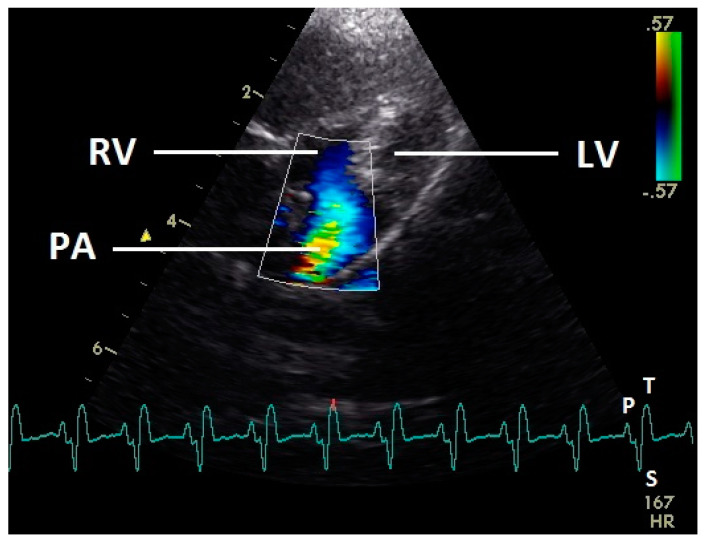
Color Doppler image of the right ventricle in the systole. The blood ejection from the right ventricle in the pulmonary artery is visible (blue signal). The blue flow signal is aliased in the pulmonary artery (wrong direction: red instead of blue of the color Doppler signal in the pulmonary artery). LV: left ventricle; RV right ventricle; PA: pulmonary artery; HR: heart rate. Electrocardiogram: P: P wave, S: S wave, T: T wave. The color scale on the right of the image is calibrated in m s^−1^.

During systole (ascending part of S wave to descending part of T wave of ECG), a blue flow signal was seen in the right ventricular outflow tract and pulmonary artery (Figure 10). In all pigeons aliasing occurred in the pulmonary artery with our settings. A regurgitation of the right AV valve was not seen.

## 4. Discussion

The blood flow in the avian heart follows the same principles as in mammals. The diastolic blood flow is characterized by an early or passive diastolic filling (E wave in pulse wave Doppler image; descending part of T wave in ECG) and an active filling during the atrial contraction (A wave in pulse wave Doppler image) or fused (EA wave in pulse wave Doppler image) in higher heart rates [3,4,10] and a systolic blood flow through the aorta and pulmonary artery (ascending part of S wave to descending part of T wave of ECG). This general blood flow was well visualized for the pigeons with color flow imaging in our study and is already described in the literature for other bird species [3,4,14,23,30]. The generally valid color coding used in small animal medicine, the diastolic blood flow color-coded in red, and the systolic blood flow in blue could also be used in the pigeons in the used acoustic windows [10]. However, faster velocities, especially in the aorta and pulmonary arteries show aliasing. In these cases, the used PRF and/or the highest PRF settings of the used ultrasound system were too low. On the other hand, blood flow of lower velocities, for example of the right atrium, is difficult to investigate and explain why these blood flows cannot be shown in all pigeons. The significant differences in the displayability of the blood flow in the areas of the septal and lateral leaflets of the left AV valve can also be explained by different velocities. These results indicate that a general setting for color Doppler echocardiography of all heart regions in pigeons does not exist and an individual manual adaptation corresponding to the region to be examined is necessary. The significant differences between the PRF settings in systole and diastole to visualize the blood flow in our investigations illustrate this necessity. An initial PRF setting can be selected according to the blood flow velocity specifications in the literature for pigeons depending on heart rate or anesthesia used [21,22,23,31].

Constant simultaneous occurrences of red and blue color in the Doppler images of ventricles and atria show the presence of typical constant blood flow vortex formations in the avian heart, comparable to the mammalian heart [32]. Vortices in the cardiovascular system are supposed to play fundamental roles in normal physiology and provide a proper balance between blood motion and the stresses on the surrounding tissues [32]. In the color Doppler examinations of the pigeon heart in the “four-chamber view”, a vortex clockwise in the left atrium and counter-clockwise in the right atrium were observed. In this context the valva pulmonis of the left atrium is discussed as an important anatomical structure for sealing the atrial cavum [1]. However, the color Doppler images in our investigation show that in diastole (atrial contraction) and systole (atrial filling) the position of the valva pulmonis is very constant. There is no evidence for a motion of this valve which could contribute to the closure of the left atrium (see Figure 5, Figure 6 and Figure 7). The main function of this anatomical structure seems to be to direct blood flow towards the left ventricle (see Figure 6). The passive filling of the atria was visible after the P wave of the ECG as a cause of the relaxation of the myocardium and in the time of the end of the T wave of the ECG as a cause of the ventricular pump mechanism by the myocardial contraction in the systole. The findings in the color Doppler sonographic examination of the ventricles allow the conclusion of the presence of an asymmetric vortex ring in the left ventricle in the passive and active ventricular filling with a stronger vortex behind the longer septal part of the left AV valve (see Figure 1, Figure 2 and Figure 3). It is possible that an asymmetric valve shape allows a typical vortex formation (larger vortex in the direction of the outflow tract) that enables a faster blood flow and emptying of the ventricle [33]. Similar vortex formations in the left ventricle are described in the mammalian heart [32,33]. In the right ventricle the active ventricular filling leads to a great counter-clockwise blood vortex in this ventricle. The blood flow behind the right AV valve in the active ventricular filling as well as the active movement of this muscular valve during the closure seem to be important for the development of this blood flow vortex (see Figure 8). In connection with the asymmetrical shape of the right ventricle and oblique arrangement of the right AV valve [1], the shape of the blood vortex simplifies the outflow of blood through the pulmonary artery.

In avian medicine the color Doppler sonography is mainly used to visualize abnormal blood flow [3,4,14]. Especially for the interpretation of a pathological blood flow the knowledge of physiological blood vortex formations could be important. However, in our study the insufficiency of the left AV valve in the preejection period could be visualized in two birds with this technique. The importance of muscle contraction of the left ventricle and the influence of anesthesia on the function of the left AV valve were previously described and discussed by Legler et al. [23]. Anesthesia with isoflurane leads to an AV valve insufficiency in the preejection period in 93% of pigeons, which was diagnosed with color Doppler sonography [23].

More research is needed to understand the complex vortex formations of blood flow in the avian heart and the physiological benefits. Furthermore, a connection between the vortex formations and distribution of the natriuretic peptides in heart muscle cells as an important part of the cardiac hormone system of birds is still unknown.

## 5. Conclusions

The diastolic and systolic blood flow as well as heart valve insufficiencies can be well visualized in pigeons with color Doppler sonography. However, the device settings must be adapted to the blood flow velocities of the region to be investigated to display the blood flow sufficiently. With the help of color Doppler imaging, typical physiological atrial and ventricular blood flow vortex formations were visualized in the avian heart for the first time. The knowledge of these physiological blood flow vortices is important to identify a pathological blood flow.
